# Targeted therapeutic options and future perspectives for HER2-positive breast cancer

**DOI:** 10.1038/s41392-019-0069-2

**Published:** 2019-09-13

**Authors:** Jiani Wang, Binghe Xu

**Affiliations:** 1 Department of Medical Oncology, National Cancer Center/National Clinical Research Center for Cancer/Cancer Hospital, Chinese Academy of Medical Sciences and Peking Union Medical College, No. 17, Panjiayuannanli, Chaoyang District, 100021 Beijing, China; 2 State Key Laboratory of Molecular Oncology, National Cancer Center/National Clinical Research Center for Cancer/Cancer Hospital, Chinese Academy of Medical Sciences and Peking Union Medical College, No. 17, Panjiayuannanli, Chaoyang District, 100021 Beijing, China

**Keywords:** Breast cancer, Drug development, Breast cancer, Drug development

## Abstract

Over the past 2 decades, there has been an extraordinary progress in the regimens developed for the treatment of human epidermal growth factor receptor 2 (HER2)-positive breast cancer. Trastuzumab, pertuzumab, lapatinib, and ado-trastuzumab emtansine (T-DM1) are commonly recommended anti-HER2 target agents by the U.S. Food and Drug Administration. This review summarizes the most significant and updated research on clinical scenarios related to HER2-positive breast cancer management in order to revise the guidelines of everyday clinical practices. In this article, we present the data on anti-HER2 clinical research of neoadjuvant, adjuvant, and metastatic studies from the past 2 decades. We also highlight some of the promising strategies that should be critically considered. Lastly, this review lists some of the ongoing clinical trials, findings of which may soon be available.

## Introduction

Breast cancer is a heterogeneous disease, which can be divided into several subtypes with diverse clinical characteristics, respective sensitivity to therapy, and different prognosis.^1,2^ Human epidermal growth factor receptor 2 (HER2) is normally overexpressed in 20–25% of breast cancers worldwide.^3^ Previous studies have established HER2 as an effective therapeutic target for the treatment of breast cancer.^4^ HER2 oncogene has been shown to play an important role in the growth and progression of breast cancer.^5,6^ Since regular updates on the clinical mechanisms and management of HER2-positive breast cancer are essential, renewed clinical practice strategies could give readers a deep insight into the potential agents and establish the framework for our daily clinical treatment.^3,7^ In this review, we summarize relevant information and enormous amount of data gathered from basic and clinical research over the past 20 years. We also highlight current clinical development that should not be overlooked. Finally, we also explore the evidence base derived from the most relevant clinical trials, which may become a part of future therapeutic options.

## Expression of HER2 and breast cancer biology

Breast cancer is the most prevalent type of malignancy in females and its risk is common, irrespective of the racial and ethnic origin of patients.^8^ Recent reports reveal that, with increased incidence, breast cancer attributes to 25% of malignancies diagnosed annually and it is the leading cause of mortality among females, worldwide.^9^ As per the routine immunohistochemical (IHC) parameters for clinic pathologic management, breast cancers are classified into four molecular subtypes^10,11^ as luminal A (ER- and/or PR-positive/HER2-negative/low Ki-67), luminal B (ER- and/or PR-positive/HER2-negative/high Ki-67), HER2-positive luminal B (ER- and/or PR-positive/HER2 overexpression/any Ki-67), non-luminal HER2-positive (ER and PR absent/HER2 overexpression), and triple negative (ER and PR absent/HER2-negative).^12^

**Fig. 1:**
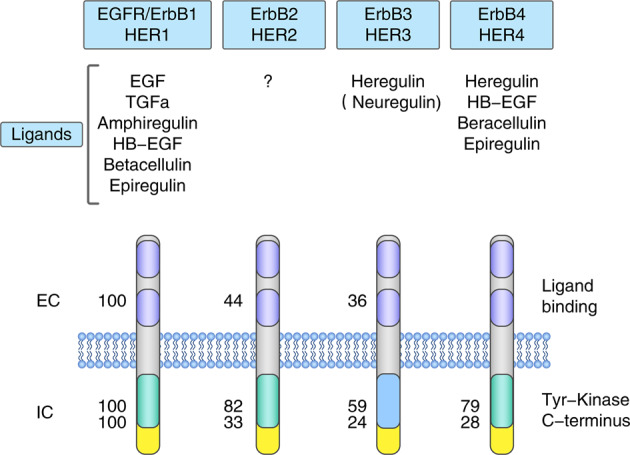
Schematic representation of human epidermal growth factor (Erb) family and ligands. Formation of different homo- and heterodimers were induced by specific ligand, triggering the recruitment of various downstream adapters, resulting in the activation of numerous signal transduction pathways. HER2/neu has no known ligands and that HER3 has no intrinsic tyrosine kinase activity

The HER2 oncogenes (HER2, HER2/neu, c-erbB-2) are located on chromosome-17^13^ and were first discovered in 1984 by Weinberg and colleagues.^14^ The main function of HER2 oncogene is to encode transmembrane receptor tyrosine kinase.^15,16^ As compared with HER2-negative tumors, HER2-positive breast cancer is aggressive subtype that demonstrates unique epidemiological, clinical, and prognostic differences with poor response to standard chemotherapy regimens.^17^ The HER2 gene amplification in the breast cancer is closely related to tumor-cell multiplication and invasion, resulting in focal progression and distant metastases.^18,19^ The amplification of HER2 genes is associated with the proliferation and progression of certain aggressive breast cells, which results from signal transduction mediated by the activation of PI3K/AKT and Ras/Raf/MEK/MAPK pathways,^19^ causing adverse biological characteristics and clinical outcomes. The human epidermal growth factor (Erb) family consist of four different receptors: EGFR (ErbB1/HER1), ErbB2 (HER2/Neu), ErbB3 (HER3), and ERbB4 (HER4) (see Fig. 1).^15,20^ Heterodimers of HER2 are more stable than other receptor non-HER2 combinations. In absence of a ligand, the HER2 proteins are capable of dimerizing with other family member, such as HER1, HER3, or HER4.^21^ HER2 acts as an important invasive biomarker with prognostic significance in advanced disease and positive-lymph node metastases.^22,23^ Overexpression of HER2 has been associated with adverse survival outcome in breast cancer.^14^ HER2 receptor expression is also related to high tumor grade mitotic count and positive-lymph node metastases.^24,25^ The HER2 gene amplification and protein overexpression is discussed in a review of 107 studies consisting of 39,730 patients, which elucidate an overall 22.2% of HER2-positive rate and a mean relative risk (RR) of 2.74 for overall survival (OS) in the anti-HER2-targeted therapy.^24^ HER2 gene amplification has been confirmed as an independent adverse prognostic factor with significance on all other prognostic variables.^26,27^

HER2-positive breast cancer may result in resistance to non-anthracycline and non-paclitaxel regimens.^28^ In addition, HER2-positive patients have a lower response rate to endocrine therapy.^29^ HER2-targeted therapies have extremely improved survival outcomes for HER2-positive breast cancer patients.^22^

## HER2 testing techniques

In clinical practice, the diagnosis of HER2-positive breast cancer is done as per “standard of practice” guidelines for the management and evaluation of breast cancers.^30,31^ In this article, the protocol for identification of HER2 in breast tumors and peripheral blood is summarized.^32^ The best method to determine HER2/neu status in breast cancer will continue to be discussed for years to come.^33^ Keeping in mind the strengths and limitations of the slide-based assays, such as IHC, fluorescence in situ hybridization (FISH),^34^ and chromogenic in situ hybridization (CISH) that are currently used, a series of morphology-driven and molecular-based techniques have been designed to detect HER2 overexpression and amplification status in breast cancer (see summary Table 1).^35–37^

**Table 1:** Techniques of HER2/neu status detection in breast cancer

Method	Samples	Target	Slide based	Utility
IHC	Tissue	Protein	Yes	A
FISH	Tissue	Gene	Yes	A
CISH	Tissue	Gene	Yes	A
SISH	Tissue	Gene	No	
RT-PCR	Tissue	mRNA	No	A
Microarray				
Tissue ELISA	Tissue	Protein	No	A
Serum ELISA	Serum	Protein	No	A
CTC	Serum	Gene	No	B

A: determining and predicting of response and eligibility to receive anti-HER2 therapy. B: monitoring response of breast cancer to anti-HER2 treatment

*IHC* Immunohistochemical, *FISH* fluorescence in situ hybridization, *CISH* chromogenic in situ hybridization, *SISH* silver in situ hybridization, *RT-PCR*, reverse transcription polymerase chain reaction, ELISA, enzyme-linked immunosorbent assay, *CTC* circulating tumor cells

### Immunohistochemistry

IHC staining is the most commonly used slide-based techniques for initial testing of HER2 status in newly diagnosed breast cancer patients. However unlike the conventional IHC assays, it is a quantitative evaluation as HER2 protein is expressed in breast epithelial cells.^28^ At present, U.S. Food and Drug Administration (U.S. FDA) has authorized two kits, Dako Hercep Test™ (Dako Corporation, Glostrup, Denmark) and Ventana Pathway™ (Ventana Medical Systems, Tucson, AZ) for making a strategic decision in determining whether the patients should undertake anti-HER2 therapy.^37^ IHC assays have been considered as the primary determining test for HER2 status and nearly 80% of initially diagnosed breast cancers patients in US had undertaken it.^38,39^ It was essential to establish a standardized IHC procedure and scoring system to provide a meaningful interpretation of a HER2 immunostaining.^40^

Standardized IHC assay has the following advantages^41^: common pathologic routine, easy slide staining techniques, wide availability, and relatively low cost; while the limitations are variation of system-control standards for storage, duration, fixation, and the difficulties of a semiquantitative and subjective slide-scoring system-based application in clinical practice.^42,43^ Studies have proved that if microscopic process, embedding, tissue process, and storage procedure are carefully performed, appropriate correlation between protein expression status and gene-copy levels can be achieved.^44^ Thus, in clinical settings, errors in HER2 testing by IHC technique arises from both, difference in correlation of antigen restoration and selection of staining reagents, and variation in pathologic slide scoring. In the United Kingdom, it has been recommended that these tests are restricted to laboratory that performs annual minimum of 250 IHC checks (and/or 100 FISH tests).^37,45^ National Surgical Adjuvant Breast and Bowel Project (NSABP) confirmed that centers undertaking high volume of HER2 testing resulted in a higher concordance between IHC and FISH outcomes.^30^ Despite the scoring system, several additional pitfalls in IHC interpretation must be expected. In order to eliminate false-positive results, pathologists must try to carefully avoid tissue injury in preparation, specimen edges scoring, cytoplasmic staining, fibrocystic metaplasia status, and intraductal (ductal carcinoma in situ) foci disease.^46,47^ Quantitative image analysis system can reduce the laboratory variability of slide scores among pathologists, which is important in routine microscopy.^48^

### Fluorescence in situ hybridization

The FISH technique done by using fluorescent-labeled probes is a morphology-driven slide-based DNA hybridization assay, to detect the HER2 gene amplification.^49^ It can utilize a chromosome-17 probe (CEP17) as an internal control.^50^ Presently, three versions of FDA-recommended FISH tests are as follows: Ventana Inform™ test (Ventana Medical Systems, Tucson, AZ), a single-probe technique that detects single HER2 gene, and the dual-probe (HER2 probe plus chromosome-17 centromere probe) kits, PathVysion™ (Abbott Laboratories, Abbott Park, IL) and PHarmDX (Dako, Glostrup, Denmark).^45^ Previous studies proved that single-probe approach is highly correlated with dual-probe test for detection results of HER2 gene status in breast cancer, suggesting that the clinical diagnostic value of the two techniques is similar,^51,52^ and the simultaneous detection of HER2 and chromosome-17 could clarify the HER2 gene status.^40,53^

From a societal perspective, FISH is an affordable objective scoring method,^54^ with the advantages of two HER2 gene signals, expressed both in benign and malignant cells.^55^ However, the limitations of FISH technique include the higher quality for slide scoring, use of fluorescent microscope, higher test cost, and more time consuming than IHC.^53^ Although still debatable, several experts strongly recommend FISH over IHC in defining the HER2 status for breast cancer, as it is more common and accurate.^44^ Generally, most of HER2 testing (80–85%) is done by IHC, and results is defined as 0 and 1+: negative, 2+: uncertain and require further FISH assay for confirmation, and 3+: positive.^45,47^ False negative FISH results are unusual, but may occur when the pathologist fails to identify the amplified areas of HER2 gene with heterogeneity.^51,52^ Thus, diligence and caution are required when scanning the case at low magnification analysis. Since the guidelines of HER2 testing from American Society of Clinical oncology (ASCO)-CAP were published,^56^ we generally considered value of 2.0 ratio for a positive FISH cutoff instead of 2.2, which resulted by the prior expert recommended.

### CISH and silver in situ hybridization (SISH)

The CISH approach and SISH method capture the advantages of both IHC and FISH.^53^ It detects HER2 gene-copy number by using a single HER2 probe. The CISH was approved by the FDA to evaluate feasibility for anti-HER2 agent.^36^ In addition, CISH has the lowest correlation with IHC 2 staining and highest with IHC 0, 1, and 3 results.^35^ Previous researches have shown about 97–99% of concordance between CISH and FISH. Several clinical trials^53^ have defined criteria as 3+ for IHC test or FISH-positive tumors; whereas others, like the Finland Herceptin (FinHer) trial, have relied on the CISH results.^57^ However, this method needs further investigations in future.

### mRNA evaluation by microarray and reverse transcription polymerase chain reaction (RT-PCR)

In breast cancer management, microarray-based mRNA measurements can assess relative levels of different mRNA molecules.^58^ Several multigene predictor assays, including Oncotype DX™, Mammaprint™, and TargetPrint™, have been approved.^59^ These assays utilize HER2 mRNA associated with other genes related to HER2 amplification, in evaluating recurrence risk of breast cancer.^60^

Relative levels of HER2 mRNA can also be detected by RT-PCR technique, although large-scale trials have not yet been conducted.^61^ This approach is relatively rapid, low cost, and has tremendous potential.^60^ Enzyme-linked immunosorbent assay (ELISA) can be used to detect the concentration of protein in extracellular domain (ECD) of tissue and serum. This technique has been approved by the FDA in the monitoring of disease, using commercially available kit like Oncogene Science HER2/neu ELISA (Oncogene Science, Cambridge, MA). Recently, a meta-analysis purposed that assessment of HER2 ECD levels in breast cancer may not be informative. Moreover, collective analysis of four trials showed that the baseline ECD level was not reliably predictive of response to the therapy.^62^ Contrarily, some studies have shown supporting data of the relationship between ECD levels and response to specific therapies.^63^ Thus, further investigation needs to be done to arrive at conclusion about the utility of HER2 ECD assessment.

### Chromosome-17 polysomy

Previous studies on invasive breast cancer have demonstrated the incidence of chromosome-17 polysomy in 4–30% of all cases.^13^ Most studies confirm the relationship between chromosome-17 polysomy and HER2 protein overexpression, which may have an effective response to trastuzumab-based treatment with restriction to IHC3+ staining cases.^50,64^ Another study proposes that chromosome-17 polysomy could be the probable cause for clinical phenomenon wherein the patients show no HER2 gene amplification, as identified by ratio-based FISH approach, but respond to trastuzumab-based treatment of metastatic breast cancer (MBC).^51^ Although not verified in large scale studies, it should be noted that in breast cancer patients with chromosome-17 polysomy, positive responses to anti-HER2-targeted therapy usually appear to be restricted to tumors with an IHC score of 3+.^40,42^

### HER2 testing of circulating tumor cells (CTC)

Tissue specimens are essential to assess HER2 status for breast cancer by IHC or FISH technique.^47^ However, numerous studies are being conducted to evaluate the HER2 status in breast cancer patients without using tissue specimens, but testing CTC.^65,66^ More research is needed to investigate the utility of CTC approach, especially in the prediction of response to therapy.^67–69^ In a metastatic scenario, it has been approved that longitudinal serum ctDNA sequencing can be done to detect drug resistance and guide the precise clinical practice for anti-HER2-targeted treatment.^70^ What is more, recent relevant researches have shown the potential of comprehensive cell-free DNA (cfDNA) detection in identifying beneficial patients from HER2-targeted therapies.^71^ A comprehensive liquid biopsy analysis that combines matched cfDNA mutations with CTC transcriptional analysis is possible to enhance the identification of operable targets for individual treatment strategies.^72,73^

## Anti-HER2-targeted agents

Intracellular and extracellular studies on the HER2 gene have provided insights into strategies to inhibit this pathway and have helped develop pharmacological anti-HER2 agents.^74^ In this review, we highlight some updated clinical concepts regarding anti-HER2 agents, which should not be overlooked in clinical practices while adhering to current conventional literature. In addition, we provide an overview of new potential agents that are currently being tested in clinical trials and may constitute an optional strategy in the near future.

### Trastuzumab therapy

Trastuzumab (Herceptin®, Genentech Corporation, United States/Hoffman-Roche, Switzerland), a monoclonal IgG1 class humanized murine antibody, binding the ECD of HER2 transmembrane receptor.^75^ It was first approved for breast cancer treatment directed against HER2.^76^

The mechanism of its antitumor action is by binding to the ECD of the HER2 receptor, including antibody-dependent cell-mediated cytotoxicity (ADCC), blockage of ligand-independent HER2 receptor dimerization.^77^ Interestingly, the inhibition of downstream signal transduction pathways and angiogenesis, induction of cell-cycle arrest and apoptosis,^78^ and interference with DNA repair, have also been confirmed as its mechanism in anti-HER2 therapy.^78–80^

Since its launch in 1998, trastuzumab became a therapeutic for breast cancer patients with HER2 overexpression and is widely administrated as approved indications in both the adjuvant and metastatic situations with the same recommended dosage.^81^ Trastuzumab is typically given by intravenous perfusion weekly or every 3-week cycles, at a dose based on body weight.^75^

Trastuzumab improves survival endpoints and quality of life for patients with advanced HER2-positive breast cancer undergoing chemotherapy.^75^ Trastuzumab was the first target approved specifically for early stage HER2-positive breast cancer in combination with cytotoxic agents, such as taxane, after completion of doxorubicin therapy.^82,83^ In addition, trastuzumab is recommended by both St. Gallen and National Comprehensive Cancer Network (NCCN) guidelines for use as a single-agent regimen monotherapy after adjuvant treatment.^84,85^ Trastuzumab, in addition to chemotherapy (either anthracycline plus cyclophosphamide or taxane agents), has gained favor in clinical practice.^86,87^ Combinations of trastuzumab with other agents have been associated with a longer disease-free progression (median, 7.4 vs 4.6 months; *p* < 0.001), a longer survival duration (median survival time, 25.1 vs 20.3 months; *p* < 0.01), a lower 1-year mortality rate (22% vs 33%; *p* < 0.008), a higher rate of objective response (50% vs 32%; *p* < 0.001), and a longer response duration (median, 9.1 vs 6.1 months; *p* < 0.001).^88,89^

In the adjuvant, neoadjuvant, or metastasis settings, a number of adverse events (AEs) have been linked to the use of trastuzumab, including acute cardiac toxicity in congestive heart failure (CHF),^90,91^ gastrointestinal symptoms, minor hematologic deficiencies, and pulmonary symptoms.^92,93^ The severe toxicity incidence rate is about 1% as per recent evidence, among which cardiac toxicity has been the most predominant limiting factor for the use of trastuzumab.^94,95^ Earlier published adjuvant clinical trials report grade 3–4 cardiac toxicity (1–4%) in trastuzumab-treated patients. Fortunately, such cardiac dysfunction is reversible through the removal of dose accumulative effects.^96^ Currently, there are no confirmed biomarkers that can precisely predict the occurrence of cardiac toxicity after trastuzumab use.^95^ However, prior or concomitant use of anthracycline and trastuzumab agents may result in a high risk of dose-dependent irreversible cardiac damage in HER2-positive patients. Finally, strategies for preventing left ventricular ejection fraction (LVEF) deterioration have been explored, which include new combinations with other cytotoxic agents (vinorelbine, taxanes, and platinum) to avoid anthracycline use.^97,98^ In NSABP B-31 adjuvant trial, abnormal LVEF, primary hypertension, history of diabetes mellitus, and advanced age were found to be significant predictive factors for CHF development.^87^

### Pertuzumab

**Fig. 2:**
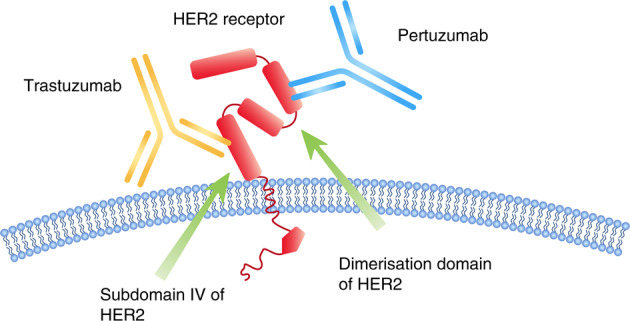
Trastuzumab and pertuzumab bind to different regions on HER2. Trastuzumab suppresses HER2 activity but does not inhibit heterodimerization. Pertuzumab has the capability of binding to the extracellular dimerization subdomain of the HER2 receptor, reducing HER2 intracellular signaling events by blocking heterodimerization with other HER receptors

Pertuzumab (Perjeta®, Genentech, United States/Hoffman-Roche, Switzerland), a humanized recombinant monoclonal antibody, prevents heterodimerization of HER2 with HER3 by interfering with the ligand-dependent HER3 mediated signaling pathway,^99^ thus inhibiting the proliferation. This is done by inactivating multiple downstream signaling networks including the phosphoinositide 3-kinase (PI3K/AKT/mTOR) and the mitogen-activated protein kinase (RAS/RAF/MEK/ERK) pathway.^100^ Complementary to trastuzumab, pertuzumab triggers an ADCC reaction and binds HER2 at a different ECD than trastuzumab (see Fig. 2).^101^

Although pertuzumab monotherapy has only shown modest anti-HER2 efficacy, there may be a synergistic effect when it is combined with trastuzumab.^102^

Pertuzumab may also be effective in cases of normal levels of HER2 with high HER1 (EGFR) levels, or breast cancers with characteristics of low-level HER2 overexpression.^101,102^ So far, efficacy of pertuzumab for HER2-positive metastatic disease has been successfully confirmed in clinical trials. On 8 June 2012, the FDA approved the combination of pertuzumab with trastuzumab and docetaxel, as first-line treatment in HER2-positive MBC.^100^ CLEOPATRA study describes a 40% ORR rate with multiple complete and partial responses.^103^ Furthermore, pertuzumab was the first drug to be approved with an endpoint of pathological complete response in the neoadjuvant chemotherapy on 30 September 2013.^104^ Interestingly, the value of pertuzumab beyond progression needs to be properly studied. Furthermore, new pertuzumab-based regimens are under investigation to improve the toxicity profile and efficacy of the available treatment.^101,102^

### Novel tyrosine kinase inhibitors (TKIs)

Several TKIs are in adjuvant clinical research for the treatment of HER2-positive early breast cancer (EBC).^105^ Various HER1/HER2 TKIs, pan-HER TKIs, and dual HER2/VEGF TKIs are in different stages of advance clinical practice.^15^

### Lapatinib

**Fig. 3:**
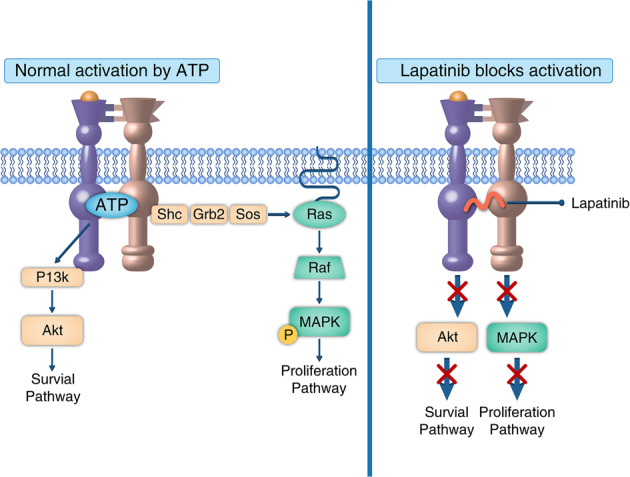
Mechanism of lapatinib action. By competing with ATP, small-molecule TKI Lapatinib blocks HER2 signaling, preventing auto phosphorylation and subsequent downstream signaling events

Lapatinib (Tykerb^TM^, GlaxoSmithKline, NC, US) is the only intracellular blocker approved for both HER2 and EGFR receptors simultaneously, achieving greater overall inhibitory effects.^106,107^ It acts as a dual reversible TKI for both these receptors, thus blocking the downstream MAPK/Erk1/2 and PI3K/AKT pathways (see Fig. 3).^108,109^ Lapatinib has been shown to enhance the trastuzumab-dependent cell-mediated cytotoxicity against breast tumor cells, in in vitro studies.^110^ Lapatinib is metabolized by the cytochrome P450 system, via the 3A4 isozyme, leading to a single metabolite activity against EGFR, without involving HER2.^111^

Lapatinib is specially approved for patients with HER2-positive advanced-stage breast cancer showing synergistic activity when combined with anti-HER2 antibodies like trastuzumab.^112,113^ A preclinical study showed that lapatinib inhibited the growth of HER2-positive breast cancer cells that were resistant to trastuzumab and increased the apoptotic effect of anti-HER2 antibodies.^114^ This suggests that lapatinib alone may be efficient for treating HER2-positive patients that are resistant to trastuzumab.^115^ The efficacy of lapatinib seems to be limited to patients exhibiting overexpression of HER2, as with trastuzumab.^116^ The combination of lapatinib and capecitabine showed a significant efficacy compared with capecitabine alone in metastatic settings.^117^

Lapatinib has an ability to diffuse in the central nervous system (CNS),^118^ thus potentially improving the control of CNS diseases as compared with the other monoclonal antibodies.^119^ Randomized trials have shown that involvement of CNS may be reduced by lapatinib administration with chemotherapy.^120^ Several trials have demonstrated that lapatinib and trastuzumab combination had better efficacy than lapatinib alone in disease progression of patients resistant to trastuzumab.^115^ Biomarkers to check the resistance to lapatinib was approved after first-line trastuzumab treatment, although less information has been published about it.^121^

A comprehensive analysis of the clinical trials featuring lapatinib in combination with various agents in MBC treatment have shown that the most frequent adverse events were diarrhea (65%), erythro-sensory disturbance (53%), nausea (44%), skin rash (28%), vomiting (26%), and fatigue (23%).^110^ However, the incidence of cardiac toxicity seems to be lower with lapatinib than trastuzumab.^122^ A pooled analysis of cardiac function in 44 studies of 3689 patients treated with lapatinib found low levels of cardiotoxicity.^122^ Cardiac events were usually asymptomatic and caused reversible decreases in LVEF. Overall, the incidence of LVEF decline was 1.6%, with 0.2% patients presenting symptomatic CHF.^123^ At present, no confirmed plasma- or tissue-based biomarkers have strongly been proven for prediction of AEs associated with lapatinib exposure.^124^

### Afatinib

Afatinib, as an oral small molecule, irreversibly inhibits HER1, -2, and -4 receptors.^125^ A phase II study in trastuzumab-resistant metastatic patients showed partial response in patients with progressive HER2-positive breast cancer.^126^ The most frequent AEs related to Afatinib include diarrhea and rash. Another phase II trial has evaluated the efficacy of afatinib, with or without vinorelbine in patients with inflammatory or MBC (NCT01325428).^127^ LUX-breast 1 is a phase III trial of afatinib or trastuzumab added to vinorelbine in patients with MBC who have received initial chemotherapy plus trastuzumab regimen (NCT01125566).^128^ However, the promising character of afatinib as a single agent or in combination with other anti-HER2 therapy is needed to be investigated further.^129^

### Neratinib

Neratinib is an irreversible pan-HER (HER1, -2, and -4 receptor) and EGFR TKI that inhibits PI3K/Akt and MAPK signaling pathways after HER2 receptor activation.^130^ Earlier clinical studies have demonstrated the use of neratinib in HER2-positive patients exposed to prior regimens of trastuzumab or anti-HER2 treatment.^131,132^ Compared with lapatinib, neratinib has been shown to have more valid and consistent inhibitory effect in feasible resistance pathways.^130^

Since neratinib and trastuzumab have different mechanisms of action on the HER2 pathway, their combination could be a synergistic strategy, as evaluated in a phase I/II MBC study.^133,134^ This trial demonstrated a promising ORR of 27% in 45 MBC patients with trastuzumab resistance. Another phase II study showed potential clinical efficacy with excellent tolerance and feasibility of neratinib.^135^ No dose limiting toxicities were recorded. Diarrhea (20%) was the most common grade 3–4 adverse events (AEs), while neutropenia (9%) and dehydration (2%) were other frequent neratinib-related grade 3 AEs. All AEs were reversible and manageable with dose reduction, pause interruption, and proper supportive care.^135,136^ Lastly, neratinib plus vinorelbine regimen was evaluated in a phase I/II clinical trial in patients initially treated with trastuzumab or lapatinib and ORR of 42% in lapatinib-treated and of 51% in lapatinib-naive patients was reported.^134^ Neratinib monotherapy is currently tested in an open-label phase II trial in HER2-positive patients with brain metastasis (NCT01494662).^137^ The earlier promising results encouraged the researchers to conduct phase III clinical studies of neratinib. A phase III trial (extended adjuvant treatment of breast cancer with neratinib, ExteNET) showed that neratinib significantly improved 5-year invasive disease-free survival (iDFS) in EBC patients, who completed trastuzumab-based adjuvant therapy.^133,138^ A combination of doublet anti-HER2 therapy, i.e., neratinib and trastuzumab plus paclitaxel, was evaluated in a phase I/II study.

### Pazopanib

Pazopanib hydrochloride is a novel multitarget receptor TKI of VEGF receptors 1, 2, 3, PDGF receptor a/β, and cytokine receptor c-kit that inhibits tumor growth and inhibits angiogenesis.^139^

A phase II study comparing lapatinib (1500 mg) in combination with either placebo or pazopanib (800 mg) in patients with relapsed HER2-positive inflammatory breast cancer (IBC) was reported at 2012 ASCO annual meeting. Significantly higher incidence of toxicity grades, i.e., 3–4 diarrhea was found in combination arm (71%) vs control arm (24%), and this cohort was terminated ahead of its expected time.^140^ No clinical benefits were reported in the combination, compared with lapatinib alone. In practice, large incidence of toxicities with the combination has been observed, especially in high-dose group of pazopanib.

### Pyrotinib

Pyrotinib, as a new generation of the HER2 targeting drug, is a small-molecule novel irreversible Pan-ErbB receptor TKI.^141^ Pyrotinib covalently binds to ATP-binding sites in the intracellular kinase region of HER1, HER2, and HER4. This inhibits the formation of HER family homodimer, blocks the activation of downstream signaling pathways, and inhibits the growth of tumor cell.^17,142^ The progression-free survival (PFS) was up to 18.1 months, as seen in its groundbreaking phase II clinical results.^142,143^

### Trastuzumab conjugates

Ado-trastuzumab emtansine (T-DM1) (Kadcyla®, Genentech, United States/Hoffman-Roche, Switzerland) is an immunoconjugate of trastuzumab with an effective microtubule inhibitor agent, which is a derivative of fungal toxin emtansine (DM1).^144,145^ This molecule has three capabilities, anti-HER2 function of trastuzumab, DM1 induced cytotoxicity, and tissue specific expression.^146^

Phase I/II studies have demonstrated good tolerance, considerable ORR, and improved PFS.^147,148^ A recent phase I trial confirmed objective responses to trastuzumab-maytansine (T-DM1) antibody conjugate (Genentech Corp., South San Francisco, CA) with the tolerated doses. To date, the KAMILLA study^149^ is the largest cohort of patients treated with T-DM1. Consistent with previous randomized studies, T-DM1 has been considered as an effective and tolerable regimen for second-line treatment of HER2-positive MBC patients.^150^

### Trifunctional antibody: ertumaxomab

The trifunctional immunoglobulin ertumaxomab (Fresenius Biotech, Hamburg, Germany) is a bispecific antibody.^151^ It targets HER2 on cancer cells and triggers T-cell-specific CD3 antigens and accessory cells, like macrophages, dendritic cells, and natural killer cells, at the sites of tumor metastases.^152,153^ Whether its molecular structure affects the immunological mechanism or induces cellular immunity against ertumaxomab is still unknown.

For multiline treated MBC patients, a phase I trial proved that ertumaxomab regimen was associated with one CR case and several PR cases.^154^ Trifunctional bivalent antibodies might complement the therapeutic efficacy of other anti-HER2/anti-ErbB receptor reagents with modular or sequential treatment.^155^

### HER2-targeted vaccines

Cancer vaccines and acquired immunity therapy targeting HER2 have been considered as leading strategies for HER2-positive breast cancer treatment.^156^ Strategies of cancer vaccines designed to produce specific anti-HER2 immunity are under research, including HER2 peptide-based vaccines, plasmid DNA-based vaccines, and vaccines with HER2 delivering in a viral vector.^157^ Active anti-HER2 immunization could facilitate the ex vivo expansion of HER2-specific T cells in adoptive immunotherapy for the treatment of MBC. Patients immunized with HER2-targeted vaccines could have strong CD8+ cell-specific responses and mediated delayed-type hypersensitivity reactions.^158^

In prior clinical trials, HER2-specific vaccines have shown efficacy and sustained levels of T-cell HER2 immunity, generating from active immunity.^159^ Evidence from prior trials have shown promising results for examining the potential use of HER2 based vaccines, in the adjuvant chemotherapy to prevent the recurrence in high-risk breast cancer patients.^160^ In a small group of patients with stage IV breast cancer, a dendritic cell-based vaccine was also been tested. One patient responded a PR, while three demonstrated stable disease (SD) profile for more than 12 months.^161^ Multiple treatment strategies were applied, including therapeutic alliance of cell-based GM-CSF secreting vaccines and trastuzumab agent. In clinical practice, normal tissues are being challenged with seldom occurrence of severe autoimmunity AEs.^158^ Future research would need to focus on developing various types of multi-epitope vaccines.^162^

### PI3K/AKT/mTOR blocking drugs

The PI3K/AKT/mTOR intracellular signal pathway regulating the cell growth and proliferation is frequently dysregulated in breast cancer and mediates primary or secondary resistance to anti-HER2 agents.^163,164^ In preclinical models, everolimus, as an mTOR inhibitor, has demonstrated efficacy in trastuzumab-resistant breast cancer.^165^

At present, two phase III trials of everolimus in anti-HER2 setting are: Breast cancer trials of oral everolimus-1^166,167^ (BOLERO-1) evaluating the combination of everolimus, trastuzumab, and paclitaxel as first-line therapy, and BOLERO-3^168^ is a randomized, double-blind, multicenter trial, which explored the efficacy of everolimus in combination with trastuzumab and vinorelbine or placebo control therapy for locally advanced or metastatic patients with prior taxane therapy. The median PFS was reported as 7.0 in the combination group vs 5.78 months in the placebo arm (*p* = 0.0067).

Two studies, BOLERO-1 and BOLERO-3, reported the combined exploratory biomarker analysis results of molecular changes in MBC with HER2 overexpression and the curative effect of everolimus.^167,168^ The final result demonstrated that patients with PIK3CA mutation, PTEN deletion, or PI3K pathway activation of HER2-positive progressive breast cancer could acquire PFS improvement from everolimus.^169,170^

### Other exploratory anti-HER2 blocking strategies

Combining anti-HER2 agents with other signaling pathway blockers could have promising progress in the future, depending on advances in clinical trials.^171,172^ Another option is the dual blockade of HER2 and non-receptor tyrosine kinase c-SRC, which plays an important role as a modulator in trastuzumab response and is a common point for multiple downstream trastuzumab-resistance mechanisms.^114^ In addition, HER3 is an another promising strong activator of the PI3K/AKT signaling pathway, which may be upregulated after the blocking of HER2.^173^

Although still in the early phases of research, the use of PI3K inhibitors and CDK4/6 inhibitors,^174^ a host of new pan-HER inhibitors, drug antibody conjugates, and anti-HER antibodies, may be clinically effective and push the boundaries further to treat HER2-positive patients in the coming years.^175,176^

## Anti-HER2 regimens

In this section we will outline salient points of clinical trials that establish the theoretical framework to guide our daily clinical practice. For convenience purpose, we classified this section into subgroups in terms of the clinical setting: adjuvant, neoadjuvant, and metastatic disease. Also, given the volume of information available in this regard, we mainly concentrated on phase III, and some phase II clinical trials. Tables 2–4 show the most important published clinical trials. Most patients were treated with regimens consisting trastuzumab and chemotherapy agents, while trastuzumab is also used as a monotherapy.^2^

**Table 2:** Selected clinical trials in the adjuvant setting for HER2 positive breast cancer

Drug or study name	Population included	No. of patients	Comparison	Median follow-up	DFS	OS	CHF/drop LVEF	Ref.
NSABP B-31/NCCTG N9831	LN (+) or high risk LN (−)	2018 973 1055	AC → T vs AC → T → H (52 weeks) vs AC → TH (H then 40 weeks)	8.4 years	62.2% (10 years) 73.7% (10 year)	75.2% (10 years) 84.0% (10 years)	0.1%/0.2% 1.5%/3.6%	Perez et al.^86,87^
HERA	LN (+) or high risk LN (−)	1703 1701 169	Std QT → H (52 weeks) vs Std QT → H (104 weeks) vs Std QT → observation	11.0 years	69.3% (10 years) 69.3% (10 years) 63% (10 years)	81% (10 years) 81% (10 years) 76% (10 years)	1.0%/4.4% 0.8%/7.3% 0.1%/0.9%	Goldhirsch et al.^188^
FINHER	LN (+) or high risk LN (−)	58 58 54 61	Docetaxel → FEC vs vinorelbine → FEC vs docetaxel + H → FEC vs vinorelbine + H → FEC	5.2 years	74.1% (5 years) 72.0% (5 years) 92.5% (5 years) 75.2% (5 years)	82.0% (5 years) 82.8% (5 years) 94.4% (5 years) 88.4% (5 years)	1.7%/10.5% (QT only) 0.9%/6.8% (QT + H)	Joensuu et al.^190^
BCIRG 006	LN (+) or high risk LN (−)	1073 1074 1075	AC → docetaxel vs AC → docetaxel + H vs TCH	10.3 years	67.9% (10 years) 73.0% (10 years) 74.6% (10 years)	78.7% (10 years) 83.3% (10 years) 85.9% (10 years)	0.7%/11.4% 2.0%/18.7% 0.4%/9.2%	Slamon et al.^186^
PACS 04	LN (+)	260 268	FE100C or ED75 → obser vs FE100C or ED75 → H	62 months	77.9% (3 years) 80.9% (3 years)	96.0% (3 years) 95.0% (3 years)	0.3%/14.2% 1.5%/35.4%	Spielmann et al.^187^
PHARE	HER-2 (+) early breast cancer	1690 1690	Std QT → H (26 weeks) Std QT → H (52 weeks)	42.5 months	91.1% (2 years) 93.8% (2 years)	96.1% (2 years) 94.5% (2 years)	5.7% (both) 1.9% (both)	Pivot et al.^196^
APT study	LN (−) and Tumor size ≤ 3 cm	406	TH × 12 weeks→ H (52 weeks)	48.2 months	93.3% (7 years)	99.2% (7 years)	0.5%/3.2%	Tolaney et al.^210^
ALTTO	HER-2 (+) early breast cancer	2093 2091 2100 2097	Std QT + L + H vs Std QT + L→ H vs Std QT + L alone vs Std QT + H alone	54.2 months	88% (7 years) 87% (7 years) 86% (7 years)	N/A	≤1%	McCullough et al.^215^
Pertuzumab (P) APHINITY	LN (+) or high risk LN (−)	2400 2405	Std QT + P + H vs Std QT + H	45.4 months	94.1% (3 years) 93.2% (3 years)	98.8% (3 years) 98.9% (3 years)	0.7% 0.3%	von Minckwitz et al.^101^
Lapatinib (L) TEACH	Stage I-IIIc H naïve 1230	1230 (HER-2+) 1260 (HER-2+)	Std QT → L (52 weeks) vs Std QT → observation	47.4 months 48.3 months	87.0% (4 years) 83.0% (4 years)	94.0% (4 years) 94.0% (4 years)	3.0% (both) 3.0% (both)	Goss et al.^212^
T-DM1 KATHERINE trial	HER2-positive EBC with residual tumor lesions	743 743	T-DM1 vs H	41.4 months	88.3% (3 years) 77.0% (3 years)	89.7% (3 years) 83.0% (3 years)	0%/0%	von Minckwitz et al.^218^
Neratinib ExteNET trial	LN (+) or high risk LN (−) ± residual tumor lesions	1420 1420	H + nneratinib 1 year H + placebo 1 year	5.2 years	90.2% (5 years) 87.7% (5 years)	N/A N/A	0%/0%	Martin et al.^133^

*LN* lymph nodes, *AC* → *T* adriamycin cyclophosphamide paclitaxel, *FEC* 5-FU epirubicin cyclophosphamide, *ED* epirubicin docetaxel, *Std QT* standard chemotherapy, *H* trastuzumab, *P* pertuzumab, *L* lapatinib, *T-DM1* trastuzumab-maytansine, *OS* overall survival, *DFS* disease-free survival, *CHF* cardiac heart failure, *LVEF* left ventricular ejection fraction, *N/A* not available or not reported

**Table 3:** Optimal clinical trials in the neoadjuvant setting for HER2-positive breast cancer

Drug or study name	Neoadjuvant chemotherapy	No. of patients	Pathological complete response (%)	Comments	Ref.
Trastuzumab (H) MD Anderson (MDACC)	T → FEC vs T → FEC + H	19 vs 23	26.3% (95% CI, 9–51%) vs 65.2% (95% CI, 43–84%)	II–IIIA, probably first study of H emphasizing better Pcr	Buzdar et al.^223^
The NOAH trial	A + T → T → CMF vs A + T → T → CMF + H	117 HER2 (+) vs 118 HER2 (+)	22% (95% CI, not reported) vs 43% (95% CI, not reported)	T3N1/T4, or N2/N3 not originally designed to test neoadjuvant setting	Gianni et al.^232^
The TECHNO trial	EC → TH	217	38.7% (95% CI, 32–45%)	Tumor size ≥ 2cm or inflammatory breast cancer suggest pCR correlate with DFS	Untch et al.^230^
The Z1041 trial	FEC → TH vs TH → FEC + H	138 vs 142	56.5% (95% CI, 48–65%) vs 54.2% (95% CI, 46–62%)	Tumor size ≥ 2cm or lymph node positive; concurrent use of H with anthracyclines is not better	Buzdar et al.^231^
The HannaH trial	Do + H (SQ) → FEC + H Do + H (IV) → FEC + H	260 vs 263	45.4% (95% CI, 39–52%) vs 40.7% (95% CI, 35–47%)	H can be administered subcutaneously	Ismael et al.^237^
The GETN(A)-1 trial	TCbH	70 patients	39% in the 27 of 70	II/III, noninflammatory, operable breast cancer	Coudert et al.^239^
Lapatinib(L) ± (H) The GeparQuinto trial	ECH → TH vs ECL → TL	309 vs 311	30.3% (95% CI, 25–36%) vs 22.7% (95% CI, 18–28%)	cT4/cT3, HR−, or HR+ (cN+; for cT2) or pNSLN+ (for cT1) lapatinib less effective than H	Untch et al.^241^
The NeoALTTO trial	TH vs TL vs THL	149 vs 154 vs 152	29.5% (22–37%) vs 24.7% (22–37%) vs 51.3% (43–59%)	Suggested that combination H + L could be quite effective	Baselga et al.^242^
The NSABP B-41 trial	AC → TH or TL or THL	181 vs 174 vs 174	52.5% (50–59.5%) vs 53.2% (45–60%) vs 62.0% (54–69%)	H + L no better. All patients received anthracyclines	Robidoux et al.^246^
Pertuzumab (P)	Do + H vs Do + P + H	107 vs 107 vs 107 vs 96	29.0% (21–38.5%) vs 45.8% (36–56%) vs 24.0% (16–34%) vs 16.8% (10–25%).	≥T2; Combination P + H result in better pCR	Gianni et al.^249,250^
The NeoSphere trial	vs Do + P vs P + H				
The TRYPHAENA trial	FEC + HP → Do + HP vs FEC → Do + HP vs TCHP	223 patients in total	62% vs 57% vs 66%	TCH + P is an active combination	Schneeweiss et al.^104^
The CHERLOB trial	T →FEC + H vs T → FEC + L vs T →FEC + HL	121 patients	25% (90% CI, 13.1–36.9%) vs 26.3% (90% CI, 14.5–38.1%) vs 46.7% (90% CI, 34.4–58.9%)	Superiority of dual HER2 neoadjuvant treatment	Guarneri et al.^256^
The KRISTINE trial	T-DM1 + P vs TC + P + H	223 vs 221	55.7% vs 44.4% (95% CI, −20.5 to −2.0)	Traditional neoadjuvant chemotherapy plus dual HER2-targeted blockade	Hurvitz et al.^251^

*T* *paclitaxel*; *F* 5-FU, *E* epirubicin, *C* cyclophosphamide, *A* adriamycin, *M* methotrexate, *Do* docetaxel, *TC* docetaxel plus carboplatin, *H* trastuzumab, *P* pertuzumab, *L* lapatinib, *T-DM1* trastuzumab-maytansine

**Table 4:** Selected clinical trials in metastatic human epidermal growth factor receptor 2 positive breast cancer

Drug or study name	Population included	No. of patients	Comparison	Median OS (mo)	Median TTP (mo)	ORR	Ref.
H0648g trial Anti-HER2 + QT H + paclitaxel (T)	Phase III, first line, MBC	469	QT (AC or T) + H vs QT	25.1 vs 20.3	7.4 vs 4.6	50% vs 32%	Slamon et al.^89^
M77001 trial	Phase II, first line, MBC	186	Do + H vs Do	31.2 vs 22.7	11.7 vs 6.1	61% vs 34%	Marty et al.^92^
US oncology	Phase III, first line, MBC	196	TCH vs TH	41.5 vs 30.6	10.7 vs 7.1	57% vs 36%	Robert et al.^269^
CHAT trial	Phase II, first line, MBC	222	Do + X + H vs Do + H	N/A	17.9 vs 12.8	71% vs 73%	Wardley et al.^268^
Single agents trastuzumab (H)	Phase II, first line, MBC	114	None-2 doses of H used	24.4	3.8	26% (18–34%)	Vogel et al.^85^
Ado-trastuzumab	Phase II, refractory MBC	110	None	N/A	6.9 (4.2–8.4)	34% (26–44%)	Krop et al.^148^
Dual anti-HER2 CLEOPATRA	Phase III, first line, MBC	808	Do + H + P vs Do + H	56.5 vs 40.8	18.5 vs 12.4	43.6% vs 30.8%	Swain et al.^103^
H + pertuzumab (P)	Phase II, failed to H	66	None-H + P single arm	N/A	5.5	24.20%	Baselga et al.^248^
EGF104900 Lap + trastuzumab	Phase III, failed to H	296	L + H vs L alone	14.0vs 9.5	2.75 vs 1.85	80% vs 69%	Blackwell et al.^284^
EMILIA trial (ado-trastuzumab)	Phase III, MBC who failed TH	991	Ado-T vs L + X	29.9vs 25.9	9.6 vs 6.4	22% vs 14%	Dieras et al.^279,280^
EGF100151 lapatinib	Phase III, failed to H	399	L + X vs X	N/A	8.4 vs 4.4	22% vs 14%	Geyer et al.^285^
TAnDEM trial anastrozole + H	Phase III, HR and HER2-positive, first line in MBC	207	Anastrozole + H vs anastrozole alone	28.5 vs 23.9	4.8 vs 2.4	20% vs 6.8%	Kaufman et al.^299^
EGF30008 trial letrozole + L	Phase III, HR and HER2 positive, first line in MBC	219	Letrozole + L vs letrozole alone	33.3 vs 32.3	8.2 vs 3.0	28% vs 15%	Curran et al.^301,302^
Pyrotinib	Phase II, ≤2 line, MBC	128	Pyrotinib + X vs L + X	N/A	18.1 vs 7.0	78.5% vs 57.1%	Binghe et al.^142,143^
BOLERO-3 trial everolimus	Phase III, MBC who failed to H	569	Everolimus + VH placebo + VH	N/A	7.0 vs 5.8	N/A	André et al.^168^

*H* trastuzumab, *P* pertuzumab, *C* carboplatin, *L* lapatinib, *V* vinorelbine, *X* capecitabine, *T* paclitaxel, *T-DM1* trastuzumab-maytansine, *QT* chemotherapy, *AI* aromatase inhibitor, *MBC* metastatic breast cancer, *HR* hormones receptor, *Do* docetaxel, *AC* adriamycin cyclophosphamide, *OS* overall survival, *TTP* time to progression, *N/A* not available or not reported

### Targeted therapeutic strategies for adjuvant setting

#### Treatment of HER2-positive EBC with trastuzumab

About 20–25% of patients with invasive breast cancer are HER2-positive and it is considered as an independent risk factor for recurrence and metastasis.^8^ Large number of data shows that trastuzumab combined with chemotherapy can reduce nearly 50% RR of recurrence and metastasis.^177^ In the adjuvant scenario, trastuzumab is the criteria for the treatment of HER2-positive patients with breast cancer.^87,178^ Despite the lack of a widely acknowledged protocol for HER2 status evaluation, these adjuvant clinical trials have explored the use of trastuzumab and yielded remarkable clinical results.^179^ Besides, central laboratory testing for HER2 status confirmation is essential before enrollment in some trials.^28^

Trastuzumab monotherapy has been recommended in several practice guidelines for high-risk patients of cardiac serious AEs, in combination with anthracyclines.^180,181^ Whether the strategy of routine trastuzumab monotherapy± endocrine therapy could reduce recurrence risk is debatable.^182^ Prior phase III trials have coherently demonstrated that trastuzumab is fundamental in adjuvant settings.^183,184^ Trastuzumab could be used in combination with anthracycline-based chemotherapy (i.e., doxorubicin and cyclophosphamide; AC), following a taxane-based regimen (paclitaxel or docetaxel) or be combined with carboplatin and docetaxel (TC).^185,186^

The FNCLCC-PACS 04^187^ was the only negative result reported in EBC adjuvant setting. Patients (*n* = 3010) were randomly assigned to anthracycline± docetaxel containing chemotherapy regimens. HER2-positive patients (*n* = 528) were subsequently randomized to undertake sequential trastuzumab treatment, every 3 weeks. The primary endpoint was DFS. However, trastuzumab-treatment arm resulted in a nonsignificant 14% reduction in relapse risk (*p* = 0.41) and no difference in OS was observed. Further analysis showed that a small portion of patients (nearly 10%) assigned to trastuzumab were confirmed treatment lose, and a quarter of patients experienced treatment pause. Moreover, sequential use appeared to be inferior as compared with the combined use.

Four significant adjuvant trials have investigated different approaches with trastuzumab as follows: Herceptin® Adjuvant (HERA),^188^ North Central Cancer Treatment Group (NCCTG) N9831, NSABP B-31^32,86,87^, and Breast Cancer International Research Group (BCIRG) 006^186^ consisting of more than 13,000 female cases with HER2-positive EBC. The NSABP B-31 compared AC-T regimen, i.e., four cycles of doxorubicin plus cyclophosphamide (AC) followed by four sequential cycles of paclitaxel every 3 weeks with AC-TH regimen, i.e., trastuzumab plus the AC-T regimen initially with paclitaxel and trastuzumab sequential administrated for 52 weeks.^88,89^ The NCCTG N9831 compared the efficacy of concurrent vs sequential administration of trastuzumab regimen, i.e., AC applied for four cycles followed by paclitaxel (T) per week for 12 cycles plus trastuzumab and the same regimen AC-T without trastuzumab.^32^ All the trastuzumab administered concurrently or sequentially to paclitaxel, for 52 weeks. The primary endpoint of these trials was DFS and the secondary endpoint was OS. Both trials were designed initially to include high-recurrence risk patients with positive axillary lymph nodes. But, the NCCTG N9831 trial also enrolled patients with node-negative disease of high-risk recurrence, defined as tumors larger than 2 cm and ER±PR-positive or tumors larger than 1 cm with negative receptors status of ER and PR.^88,89^

A joint analysis of these clinical trials presented the follow up of 2 years and 4 years data.^88,89^ The hazard ratio (HR) for DFS was 0.48 (95% CI, 0.39–0.59; *p* < 0.001) of 2 years. At 4 years, 85.3% of patients treated with trastuzumab were disease free and alive compared with 67.1% in the single chemotherapy arm, and mortality was reduced by 33%. Updated analysis results were consistent with previous observed data. The final analysis of these studies was demonstrated in 2012, San Antonio Breast Cancer Symposium (SABCS) annual meeting, as 10-year DFS of 73.7% vs 62.2% (*p* < 0.001). An OS of 84% vs 75.2% (*p* < 0.001) was also reported in trastuzumab arm.^88^ Overall analysis confirmed that trastuzumab-contained regimens contributed to a 40% reduction of recurrence risk in terms of 10-year DFS, and 37% benefits in OS. The NCCTG N9831 trial also demonstrated an improvement trend toward the DFS in the concurrent group.^89^ Although, the sequential option showed a better outcome than placebo (*p* < 0.001).

The benefit of trastuzumab was uniform among different trials, despite diversity in patient populations, chemotherapy regimens, and duration of anti-HER2-targeted therapy. These trials have validated that trastuzumab cut down the 3-year risk of relapse by about 30% in HER2-positive EBC population. There was also an OS benefit, as seen in the HERA^178^ and BCIRG trials.^32,88^ When a 52 weeks course of trastuzumab was added to adjuvant chemotherapy, the DFS improved by 33–52% and the OS time was 34–41% greater than single chemotherapy. Independent beneficial points such as age, lymph nodal status, hormonal status, or tumor size were confirmed by univariate cox regression analyses.^178,189^

A majority of phase III adjuvant trials focus on trastuzumab and different combined chemotherapy at various doses and administration strategies. The FinHer trial^190^ was aimed to compare vinorelbine and docetaxel, as adjuvant chemotherapy agents, in high-recurrence risk patients with or without lymph node positive breast cancer. Subgroup consisting of 232 patients with amplified HER2 status were randomized to receive docetaxel or vinorelbine with trastuzumab of nine weekly cycles.^57^ Overall, the result addressed that trastuzumab group had superior recurrence-free survival (RFS) (80% vs 73%; *p* = 0.12), irrespective of combination of chemotherapy drugs used. This benefit was consistent when adjusted in lymph node metastatic patients, treated with docetaxel over vinorelbine.^190–192^ However, the limitation of this trial was the short course of treatment that might have underestimated the true efficacy of trastuzumab and also the small sample size of patients with HER2-positive tumors detect a statistically significant benefit with trastuzumab with strong study power.^82,193^

Cardiac toxicity is the most rigorous AE of trastuzumab treatment, especially in combination with anthracyclines agents. Hence, there is a need to explore anthracycline-free regimens to avoid synergistic toxicities, especially cardiac AEs.^90,91^ The BCIRG 006 study was designed to deal with this situation.^186^ This phase III clinical study enrolled HER2-positive patients of high-recurrence risk to treat with AC-T regimen, i.e., doxorubicin plus cyclophosphamide followed docetaxel and AC-TH regimen (AC-T regimen plus trastuzumab) or TCH regimen (docetaxel, carboplatin combined with trastuzumab). With a median follow up of 65 months, 5-year DFS was superior in trastuzumab-containing arms: 81% (*p* = 0.04) in the TCH arm, 84% (*p* < 0.001) in the AC-TH arm, and 75% in the AC-T arm (control). Meanwhile, OS rate was also increased (92% with AC-TH vs 91% with TCH vs 87% in the AC-T; *p* < 0.001 and 0.04, respectively). The incidence of cardiac toxicity was less in TCH regimen (0.4%), as compared with AC-TH regimen (2%). More than 10% of patients experienced LVEF reductions from basic measurements, which occurred more frequently in AC-TH subgroup than TCH subgroup (18.6 vs 9.4%; *p* < 0.001). Meanwhile, the rate of symptomatic CHF favored regimens with TCH (*p* < 0.001). According to these results, anthracycline-free chemotherapy was efficient and an optional strategy in patients with high risk of cardiac toxicity, such as comorbidity of hypertension and diabetes mellitus. In follow-up with long-term cardiac safety observation, cardiac events were observed to be within acceptable levels in the trastuzumab-containing arms of previous trials.^97,98^

#### Duration of anti-HER2-targeted therapy

Furthermore, a majority of adjuvant trials delineated the relationship between treatment duration of trastuzumab regimens and difference in survival benefits. Multiple clinical studies including NASBP-31, N9831, BCIRG 006, and HERA have proved that the application of trastuzumab in the adjuvant treatment of HER2-positive EBC for 1 year can significantly improve the DFS and OS rate.^86^^,87^^,186^^,188^ Although 1-year trastuzumab duration as standard recommendation has been approved for adjuvant scenario, studies have explored the effects of different treatment durations, from 9 weeks to 2 years. Based on the 11-year follow-up of HERA trial, trastuzumab as an adjuvant therapy for HER2-positive EBC significantly improved the survival of patients. Adjuvant treatment with trastuzumab for 2 years added no benefit than 1 year, instead a rise in cardiac toxicity was observed. There was also no significant improvement in survival efficacy after prolonged trastuzumab treatment for different hormone receptor status.^188^

However, there were special investigations on whether the treatment duration could be shortened to 6 months or 9 weeks and evaluate its activity, tolerance, and cost.^194,195^ A noninferiority study, PHARE trial, was designed to evaluate the length of trastuzumab adjuvant treatment, 6 months vs 1 year.^196^ A total of 1691 patients were enrolled in 12-months subgroup, and 1693 in 6-months subgroup, after receiving at least four cycles of adjuvant chemotherapy. In subgroup analysis, patients were divided as per hormone receptor status and treatment methods, including concurrent or sequential therapy. Results from 3.5 years of follow-up confirmed that 2-year DFS was superior for the 12-months group than the 6-months group (93.8% vs 91.1%, HR =1.28; 95% CI, 1.05–1.56). It was concluded that 6-months treatment failed to meet the noninferior efficacy of trastuzumab that was seen at 12 months.^197^ However, cardiac AEs were commonly observed in the 12-month treatment (5.7% vs 1.9%; *p* < 0.001), despite this, 12 months of adjuvant trastuzumab remained a standard treatment.^96^ The Hellenic Oncology Research Group study drew a similar conclusion.^198^ A similar randomized controlled study, PERSEPHONE, may not be continued, due to the negative results.^199^

The short-course study of trastuzumab (9 weeks vs 1 year) was investigated to assess the noninferiority of adjuvant trastuzumab combined with chemotherapy as it appeared promising (NCT00629278).^195^ A total of 1254 patients were randomized into long arm (*n* = 627) and short arm (*n* = 626). The 5-year DFS was 88% in the long arm and 85% in the short arm. According to the Bayesian analysis, the probability that the short arm was noninferior to the long one was 80% (HR 1.13, 90% CI, 0.89–1.42), with the upper limit of the CI crossing the noninferiority margin. The 5-year OS was 95.2% in the long arm and 95.0% in the short arm (HR 1.07, 90% CI, 0.74–1.56). Cardiac events were significantly lower in the short arm (risk ratio 0.33, 95% CI, 0.22–0.50, *p* < 0.0001). This study failed to show the noninferiority of a shorter period of trastuzumab administration. However, a 9-week administration decreased the risk of severe cardiac toxicity and was proposed as a potential option in patients with cardiac events during treatment, and for those with a low risk of relapse. Short-HER and SOLD studies also explored the trastuzumab treatment for 12 months and 3 months in HER2-positive EBC, and no expected positive survival results were reported.^200^

Overall, the optimal duration of anti-HER2 has been under discussion and even though the answer is still disputed, the available evidence confirms that 1-year adjuvant trastuzumab treatment is probably the most appropriate.^75^ Cardiac toxicity has been a major concern, since trastuzumab can result in reduction of LVEF and cause symptomatic CHF, which may be reversible by treatment interruption. As the evidence on whether shortened or prolonged trastuzumab treatment would benefit, is considerably lacking, further randomized controlled studies are needed.^124,201^

How about strategies for HER2-positive EBC with small tumor size?^202^ The NCCN guidelines recommend that EBC patients with high risk of recurrence should be treated with chemotherapy combined with trastuzumab.^203,204^ For small tumor size patients lacking high-risk factors, especially for patients with T1a and T1b, the most exploring evidence comes from retrospective studies and meta-analysis.^205–207^ Some retrospective studies have found that patients with T1a and T1b have good prognosis, although the benefits of chemotherapy and targeted therapy are not obvious.^182,208^ A meta-analysis published in 2015 collected data from five large randomized clinical trials^209^: HERA, NCCTG N9831, NSABP B-31, PACS 04, and FinHER. A total of 4221 patients with tumor size less than 2 cm were enrolled to analyze the benefit of anti-HER2-targeted therapy. The results showed that the 8-year cumulative incidence of DFS events was significantly lower in targeted therapy than in the control group (17.3% vs 24.3%, *p* < 0.001). Prospective clinical trial, APT study, was the only nonrandomized, single-arm trial attempting to reduce chemotherapy and target therapy for low-risk patients, with lymph node negative and tumor diameter less than 3 cm. The results showed that the regimen of 1-week paclitaxel plus trastuzumab for 12 cycles, and sequential trastuzumab for 13 cycles could reach an appreciable 7-year DFS as 93.3%. This study provided a promising option for our clinical practice, although evidence was insufficient for change in treatment decisions.^210^

#### Other strategies for the HER2-positive EBC treatment

Lapatinib is normally approved for second-line MBC, however its use in the adjuvant setting could be promising, as it is orally taken.^211–213^ The Tykerb evaluation after chemotherapy (TEACH) trial discussed the efficacy of lapatinib as adjuvant treatment in trastuzumab-naive patients. A total number of 3147 patients were randomized into lapatinib or placebo groups for 52 weeks of treatment, or until events of disease progression or death. However, DFS with lapatinib was prolonged nonsignificantly (87% vs 83%; *p* = 0.09). For subgroup analysis, patients with centrally confirmed HER2 status the HR was 0.92 (*p* = 0.94). It was seen that the lapatinib monotherapy had low efficacy in the adjuvant setting for EBC.^212,214^ Another trial named “the adjuvant lapatinib and/or trastuzumab treatment optimization” (ALTTO),^215^ sponsored by the National Cancer Institute (USA), enrolled more than 8000 patients for the evaluation of the efficacy of lapatinib combination with trastuzumab regimens, and lapatinib sequential to trastuzumab regimens, vs trastuzumab alone. The median follow-up time was 4.5 years. Compared with trastuzumab monotherapy, patients with lapatinib plus trastuzumab who were treated sequentially or concurrently had a lower risk of iDFS, without significant difference. The DFS rates were similar in the three treatment groups (86% in trastuzumab group, 88% in lapatinib + trastuzumab group, and 87% in the sequential treatment group). Compared with trastuzumab monotherapy, the incidence of AEs in combination therapy was higher, and the incidence of severe cardio-related AEs was extremely low. The incidence of CHF was less than 1%, even in 95% of patients treated with anthracycline chemotherapy. Compared with trastuzumab monotherapy, lapatinib + trastuzumab had no significant advantage in the treatment of HER2-positive EBC. Based on these evidences, lapatinib was not recommended for adjuvant therapy.^212,214,216^

Pertuzumab when added to adjuvant trastuzumab and chemotherapy improves the outcomes of patients with HER2-positive EBC.^101^ APHINITY clinical trials randomly enrolled patients with high-recurrence risk in HER2-positive EBC.^217^ Pertuzumab or placebo was administrated to adjuvant chemotherapy in addition to standard 52 weeks trastuzumab treatment. The results showed that 3-year iDFS rate was significantly improved in the pertuzumab arm, as 94.1%, compared with 93.2% in the placebo arm, with HR of 0.77 (95% CI, 0.62–0.96; *p* = 0.02).^101^ In safety analysis, previous reported AEs, such as CHF, cardiac dysfunction, and cardiotoxicity related mortality, were seldom reported in both the treatment groups.^101^ Diarrhea of grade 3–4 was reported during chemotherapy, with higher incidences in pertuzumab arm than placebo (9.8% vs 3.7%).

KATHERINE study, a phase III, open-label clinical trial compared T-DM1 vs trastuzumab as adjuvant therapy, for 14 cycles in HER2-positive EBC patients with residual invasive disease, after standard neoadjuvant chemotherapy of anti-HER2-targeted therapy, including trastuzumab.^218^

For HER2-positive EBC with residual tumor lesions (breast and/or axillary lymph node invasive carcinoma) after neoadjuvant therapy, T-DM1 treatment at the adjuvant stage significantly reduces the risk of disease recurrence or death by 50% compared with trastuzumab.^218,219^ The 3-year iDFS was 88.3% in the T-DM1 arm and 77.0% in the trastuzumab arm. The iDFS was significantly higher in the T-DM1 arm than in the trastuzumab (HR 0.50; 95% confidence interval, 0.39–0.64; *p* < 0.001). The safety data of T-DM1 was consistent with earlier studies, and no new AE risks was reported.^220^

### Targeted therapeutic strategies for neoadjuvant treatment

#### Trastuzumab in neoadjuvant therapy

HER2-positive breast cancers may have potential chemosensitivity in combination with trastuzumab, in the neoadjuvant treatment.^221^ The trastuzumab treatment in neoadjuvant therapy provides significant clinical benefits and reduces the rate of distant metastasis.^222^ The HER2 gene amplification is shown to be related to the growth and survival of breast cancer stem cells, to some extent.^223,224^

As seen in both, completed and ongoing clinical studies, the trastuzumab-based neoadjuvant therapy has a higher pathologic complete response (pCR, defined as the absence of residual cancer in breast or axillary lymph node pathology)^224^ in the treatment of HER2-positive breast cancer.^225^ Such phenomena results from removing or downregulating HER2-mediated growth signals to inhibit stem cell proliferation and invasion.^226^ There is need to explore about HER2-targeted therapy that can convert a HER2-positive into a HER2-negative subtype.^227^ As per the previous reports, to achieve pCR in neoadjuvant setting of trastuzumab plus chemotherapy, nearly 33% of patients with HER2 overexpression were converted to HER2-negative subtype in the treatment failure group.^228^

There are still some limitations in this assumption, and it requires further discussion and additional prospective studies to validate.^229,230^

The treatment with trastuzumab could improve the possibility of achieving pCR.^226^ A small randomized trial conducted by the MD Anderson cancer group was perhaps the first study confirming the role of anti-HER2 therapies in the neoadjuvant scenario.^223,231^ Although only 42 cases were enrolled, the competition of trastuzumab to sequential paclitaxel chemotherapy of four cycles followed by FEC of four cycles regimens resulted in an outstanding high rate of 2.5 times (66.7%) of pCR, than chemotherapy-alone arm (25%), *p* = 0.02. Despite the small sample size, the updated versions of this study also confirmed the findings.^231^

A multicenter, open-label, randomized phase III study, NOAH trial, was designed to add further enthusiasm to neoadjuvant anti-HER2 approach.^232^ This trial enrolled women with locally advanced or inflammatory HER2-positive breast cancer, to compare neoadjuvant chemotherapy plus trastuzumab, followed by standard 52 weeks trastuzumab maintenance, vs only neoadjuvant chemotherapy. With a median follow-up of 5.4 years, neoadjuvant treatment with trastuzumab improved the 5-year event-free survival rate as 58% (95% CI, 48–66) to 43% (*p* < 0.001), with an unadjusted HR of 0.64 (95% CI, 0.44–0.93; two-sided log-rank, *p* = 0.016). A strong association with pCR was observed in patients given trastuzumab, improving the pCR from 22 to 43% (*p* < 0.001).^233^ These results provided new insight into the association between pCR and survival outcomes in HER2-positive disease.^234^ Trastuzumab also resulted in a 40% RR of relapse, progression, or mortality compared with chemotherapy alone, with HR was 0.29 (95% CI, 0.11–0.78) for sustained survival benefit, between those with and without trastuzumab.^232,233^

In addition, TECHNO trial,^230^ an open label, phase II study demonstrated results of 217 HER2-positive patients with characteristics of larger tumors (≥ 2cm), who received four cycles of AC (epirubicin and cyclophosphamide, EC), followed by four cycles of TH (paclitaxel and trastuzumab) as neoadjuvant treatment. Overall, pCR was accomplished in nearly 38.7% and 3-year DFS (88% vs 71%; *p* = 0.003) and OS (96% vs 85%; *p* = 0.007) was improved.

Meanwhile, other trials, such as the American Z1041 trial,^231^ GeparQuattro study,^235,236^ and HannaH trial,^237^ also enrolled HER2 breast cancer with similar inclusion criteria as TECHNO to evaluate treatment with chemotherapy plus trastuzumab as concurrently or consequence regimens. A high proportion of pCR range from 32 to 56% was acquired. However, the combination of anthracyclines and trastuzumab regimen resulted in higher incidences of cardiac toxicity (2.9% vs 0.8% at 12 weeks), respectively.^238^ Trastuzumab combined with docetaxel and carboplatin achieved good pCR rate and tolerance for stage II and III HER2-positive breast cancer in trastuzumab-based neoadjuvant therapy, resulting from the GETN (A)-1 trial.^239^

#### Other target therapy in neoadjuvant setting

In order to achieve higher efficacy of anti-HER2 therapies, several randomized phase III studies have explored the hypothesis of lapatinib monotherapy or its addition to trastuzumab regimen.^240^

The phase III study of neoadjuvant therapy GeparQuinto compared the efficacy of two HER2-targeted drugs, lapatinib and trastuzumab, with the combination of four cycles of chemotherapy with EC, followed by docetaxel. The result confirmed that trastuzumab arm showed ~7% more pCR than lapatinib arm (30.3% vs 22.7%; *p* = 0.04).^241^ According to these results, and significant AEs observed in this study, lapatinib could not replace trastuzumab in the neoadjuvant chemotherapy; however, doublet HER2 inhibition may be a promising option. The DFS, distant DFS (DDFS), and OS of patients with pCR were significantly better than those without pCR (DFS: HR, 0.63, *p* = 0.042; DDFS: HR, 0.55; *p* = 0. 021; OS: HR, 0.31; *p* = 0.004).

The NeoALTTO trial,^242^ an international, randomized, open-label, multicenter, phase III study, compared the efficacy of lapatinib or trastuzumab monotherapy, or the concomitant lapatinib and trastuzumab regimen, in addition to paclitaxel, in neoadjuvant setting.^243,244^ Promisingly, the combination arm showed a prominent progress on pCR of 51%, almost twice as much as the other two monotherapies against HER2 (29.5% in trastuzumab alone and 24.7% in lapatinib alone, *p* < 0.001). In addition, the use of lapatinib was associated with severe AEs, such as diarrhea and rash. Nevertheless, contradictory to previous NeoALTTO result, NSABP B-41 study^245,246^ showed no statistical difference between the anti-HER2 combination and lapatinib or trastuzumab monotherapy. Concomitant therapy needs further evidence to clarify. In the NSABP B-41 study, all patient received AC × 4 cycles and were then randomized in the sequential chemotherapy phase of paclitaxel to trastuzumab, and lapatinib or combination arm. However, the pCR rates between three arms were abnormally high (62% in combination, 53.5% in trastuzumab arm, and 52.5% in lapatinib arm). So far, lapatinib has not been approved by the FDA for the use in neoadjuvant therapy.^213,247^ This dual inhibitory HER2 regimen of lapatinib combined with trastuzumab in neoadjuvant therapy significantly increased the pCR rate and improved the prognosis of pCR patients. However this did not translate into survival benefits, such as DFS and OS.^242^

Nonetheless, the FDA has expedited approval of pertuzumab and trastuzumab regimen, combined with chemotherapy for neoadjuvant setting. This optional strategy was based on the following two phase II clinical trials.^248^ A randomized phase II study, NeoSphere trial^249,250^ was designed for multiple centers, in which HER2-positive patients were randomized to one of four following subgroups: pertuzumab (P) + trastuzumab (T) + docetaxel (D); T + D; P + D, or P + T. All enrolled patients underwent breast mastectomy followed by adjuvant FEC chemotherapy and routine 52 weeks trastuzumab treatment. In primary endpoint analysis (i.e., pCR, DFS, OS, etc.), the combination containing triple-agents (P + T + D) showed the statistically significant higher pCR rate (46%) than doublet arm T + D (29%) and P + D arm (24%) and the T + P arm (17%). Even without chemotherapy, dual blockage of the HER2 receptor could still acquire at least 17% pCR rate. This meant that some patients may not need chemotherapy at the stage of neoadjuvant therapy, and a simple double-targeted therapy, as an attractive, can achieve good results. Notably, the incidence of cardiac toxicity did not rise even when complimented with pertuzumab (4–5% EF drop across all groups). The TRYPHAENA trial,^104^ which contained one of its three arms as anthracycline-free regimen, achieved more than 55% pCR in double HER2 blockage of trastuzumab and pertuzumab in neoadjuvant setting. Thus, anthracycline-free regimen might be appropriate regimen for the lower risk breast cancer (small tumors with negative lymph nodes) or in patients with older age and prior cardiovascular comorbidities.

To sum up, the above study discussed and approved the current data supporting the use of anti-HER2 agents, either single or combination in neoadjuvant setting.^251^ Moreover, in all of the clinical trials, pCR has been considered as a valid primary endpoint for clinical outcome evaluation. There are still some doubts about whether pCR can transfer into survival index with DFS and OS.^252,253^ It has been established by many studies, including by the FDA, that pCR is a predictor of survival in patients for anti-HER2 therapy with localized breast cancer. A meta-analysis, SABCS, published in 2012, enrolled 12,900 patients in randomized neoadjuvant trials.^254^ The pCR was significantly associated with RFS in all the analyzed subgroups. This study confirmed that pCR was most likely to predict survival outcome.^255^

In addition, a randomized trial, CHERLOB,^256^ explored neoadjuvant chemotherapy plus trastuzumab or lapatinib or the doublets in HER2-positive breast cancer with a larger tumor size (>2 cm). The endpoint was pCR, for both of these trials that compared lapatinib plus cytotoxic agents with lapatinib plus trastuzumab plus cytotoxic agents. However, the efficacy data of these trials has not been disclosed.

A small phase II trial compared various neoadjuvant monotherapy in operable HER2-positive breast cancer for 6 weeks prior to surgery treatment. Single-agents included afatinib (50 mg/day), lapatinib (1500 mg/day), and trastuzumab (2 mg/kg weekly, after initial dose) and the results suggested that afatinib had higher RR compared with the other two agents (80% vs 75% vs 36%, respectively).^256^

The KRISTINE trail^251^ achieved significant results in chemotherapy plus dual HER2-targeted blockade (docetaxel, carboplatin, and trastuzumab plus pertuzumab). Compared with chemotherapy plus HER2-targeted therapy (T-DM1 plus pertuzumab), more patients achieved pCR (55.7% vs 44·4%, 95% CI, −20·5 to −2·0; *p* = 0·016). However, combination groups had more grade 3-4 severe adverse events. It is necessary to further improve the efficacy of chemotherapy without increasing toxicity.

To summarize, neoadjuvant with anti-HER2 agents is an effective and approved treatment option, especially in patients with locally advanced, unresectable tumors. Its use in small resectable cancer may also be appropriate, but it must be balanced with practical considerations and the patient's preferences.^226^

### Targeted therapeutic strategies for metastatic disease

HER2-positive advanced breast cancer (ABC) is an aggressive disease, associated with a poor prognosis and severe survival outcomes. HER2-positive breast cancer in the advanced settings prolongs both PFS and OS when combined with chemotherapy and it has become the standard strategy.^12,257,258^ Despite the remarkable therapeutic impact of anti-HER2 considering the previous outcomes, some HER2-positive patients may initially have primary resistant disease, causing eventual progression, and this pressing the need for other novel therapeutic options.^259^ We review recent phase III trial data and discuss a practical approach to sequencing of HER2-directed therapy in patients with HER2-positive MBC. The significant cumulative survival evidence gained from these trials is expending our insights of outcomes of HER2-positive MBC.

#### First-line treatment for HER2-positive MBC

Trastuzumab combined with chemotherapy is currently a novel recommended treatment of HER2-positive breast cancer patients.^260^ Besides, the other emerging anti-HER2 targeting drugs may serve as new standards in the future.^261^ Previous pioneering studies have led the foundation of our understanding of anti-HER2 therapies, and some new concepts should be highly valued.^176,262^

Taxanes are the agents most commonly administrated in combination with trastuzumab^263^; however, other regimens such as anthracyclines,^181^ vinorelbine,^193,264^ platinum, capecitabine^243,265^, and combination have been explored in several researches. Presently, NCCN Clinical Practice Guidelines recommends the following regimens, for the first-line options of HER2-positive MBC^266,267^: trastuzumab plus chemotherapy single agents, either paclitaxel (3 weeks or weekly cycle), docetaxel (3 weeks or weekly cycle), or vinorelbine (weekly).^268^ Meanwhile, trastuzumab plus paclitaxel and carboplatin (DCH, 3 weeks per cycle) or docetaxel plus carboplatin (TCH, every 3 weeks) have also been recommended for combination therapies.^185,269^ Combined use of anthracyclines-free regimens, especially carboplatin-based trastuzumab regimen, has been proved to be effective with higher overall RR and longer PFS time.^238^

In addition, new anti-HER2 therapies, either as monotherapy or combined with trastuzumab, have demonstrated anti-HER2 tumor activity.^270,271^ Single anti-HER2 drugs appear to be mild but with consistent activity. This has been well confirmed not only for trastuzumab monotherapies (ORR: 15–25%), but also for pertuzumab (ORR: 5%), lapatinib (ORR: 5%-7%), and T-DM1 (ORR: 35%). When first-line trastuzumab-containing regimens fails, the newer drugs may bring sustained SD, thus adding to the clinical benefits.^184,272^

Dual distinct anti-HER2 therapies could be combined to achieve synergistic effect.^171,172^ Three combinations are particularly suggestive: (1) Pertuzumab + trastuzumab + docetaxel; (2) Trastuzumab + lapatinib; and (3) Pertuzumab + trastuzumab. The CLEOPATRA study^103^ randomized 808 MBC patients with naive HER2-positive status, to either the standard regimen (trastuzumab + docetaxel) or the same combination plus pertuzumab. Almost 50% prolongation in PFS favoring the experimental arm was observed in this trial (18.5 vs 12.4 months; *p* < 0.001). A significant improvement of OS was confirmed by the updated results. Currently, triple-agents including pertuzumab, trastuzumab, and docetaxel are evaluated as the standard care for first-line treatment MBC, as per NCCN guidelines. This regimen utilizes the concepts previously mentioned, dual HER2 blockage and addition of chemotherapy drugs.

#### Strategies for HER2-positive MBC with initial trastuzumab failure

Some patients are resistant to trastuzumab and develop disease progression within 1 year, after initial treatment.^20,273^ Strategies for HER2-positive MBC with initial trastuzumab failure is a question worth exploring.

An observational Hermine study^274^ found that the median OS and time to progression (TTP) of breast cancer patients who continued to receive trastuzumab after disease progression were significantly longer than those who stopped using it. Phase III GBG26 trial^275^ compared the use of capecitabine single-agent and trastuzumab plus capecitabine, in HER2 breast cancer targeted therapy, after progression. The results showed that the ORR and TTP were superior in combination group than chemotherapy alone, and toxicity incidence was not increased. These two studies further confirmed trastuzumab as a first-line agent for MBC treatment in clinical practice.^266,276^

However, dual anti-HER2 blockage remains an option, especially in initially treated patients, having poor tolerance to chemotherapy.^172,277,278^ EMILIA study,^279,280^ a randomized, open-label, phase III trial compared T-DM1 with capecitabine + lapatinib in patients with previous treatment failure for HER2-positive unresectable MBC. These patients were previously treated with standard combination of trastuzumab plus taxane. Enrolled patients were randomized in 1:1 ratio to T-DM1 arm (3.6 mg/kg intravenously, every 21 days) or control arm (capecitabine 1000 mg/m², orally twice daily for 1–14 days; plus lapatinib 1250 mg, orally once daily on 1–21 days, 3 weeks one cycle). A descriptive analysis of final survival outcome shows that T-DM1 improved OS.^281^ Median OS was longer in T-DM1 arm (29.9 months, 95% CI, 26.3–34.1) than in control group (25.9 months, 95% CI, 22.7–28.3), with the HR of 0.75 (95% CI, 0.64–0.88) in previously treated HER2-positive MBC patients. In safety analysis, the incidence of grade 3 AEs was lower in the T-DM1 (48% vs 60%). The safety data were similar to prior results, reconfirming T-DM1 as an effective and tolerable treatment. Nowadays, T-DM1 has been wildly recognized as the preferred second-line treatment in HER2-positive MBC population resistance to initial trastuzumab.^282,283^

An open-label, phase III trial, EGF104900^284^ compared the efficacy of lapatinib monotherapy with lapatinib plus trastuzumab (median 3 prior regimens) in the treatment of HER2-positive MBC, and found improvement of PFS (11.1 weeks vs 8.1 weeks; *p* = 0.008) and clinical benefit rate of dual blockage-targeted therapy. Combination showed a trend of prolong OS (14 vs 9.5 months; *p* = 0.026) better than that of lapatinib alone. This trial confirmed the efficacy of lapatinib plus trastuzumab, as double targeting on breast cancer cells as seen in preclinical studies. Thus providing a safe and effective alternative for patients with non-chemotherapy HER2+ MBC.^244,284^

The EGF100151 study^177,285^ compared the effect of lapatinib plus capecitabine and capecitabine monotherapy as the second-line treatment in patients with advanced HER2-positive breast cancer. The results showed that lapatinib plus capecitabine significantly prolonged TTP; however, could not improve the OS.

Another promising strategy is chemotherapy-free combination of pertuzumab and trastuzumab.^286^ Baselga et al. conducted a phase II trial^248^ by using chemotherapy-free combination. This study reported a progressed ORR of 24% and a median PFS of 5.5 months for patients enrolled with failure of initial trastuzumab-based therapy.

BOLERO-3 study^168^ included patients who progressed during adjuvant treatment, or within 12 months after treatment, or who progressed within 4 weeks after trastuzumab treatment. After randomization, one group was treated with everolimus + vinorelbine + trastuzumab, and the other with placebo + vinorelbine + trastuzumab. The results showed that the median PFS was 7 months in the everolimus group, compared with 5.78 months in the placebo group.

What about third-line treatment with T-DM1 resistance in HER2-positive breast cancer?^287,288^ Anti-HER2-targeted therapy is used throughout multiline treatment. Treatment strategies include either continuing with trastuzumab, switching to trastuzumab in combination with lapatinib, or considering lapatinib and capecitabine.^289^ The mechanism of trastuzumab resistance needs to be studied further.^20,74,259^ In view of the differences in drug resistance time, more appropriate treatment strategies should be considered in order to prolong the survival of patients and improve the quality of life.^176^

A recent phase II clinical study compared combination of pyrotinib with capecitabine^17,141–143^ and lapatinib with capecitabine, to treat the patients with advanced HER2-positive breast cancer who had previously used/not used trastuzumab within two lines. The ORR of patients in the pyrotinib arm was significantly higher than that in the lapatinib arm (78.5% vs 57.1%; *p* = 0.01). Further analysis showed that the median PFS in the pyrotinib group was significantly better than in the lapatinib group (18.1 vs 7.0 months; *p* < 0.0001). Subgroup analysis showed that in the previous trastuzumab subgroup, the median PFS of the pyrotinib group was significantly better than that of the lapatinib group (18.1 months vs 7.1 months, *p* = 0.0031). It was suggested that the antitumor effect of pyrotinib is independent of earlier use of trastuzumab.^141,143^

#### Targeted strategies for triple-positive MBC

Triple-positive patients (both ER/PR-positive and HER2-positive) could also benefit from dual blockage.^290,291^ Nearly 50% of the breast cancers with HER2 amplification also have some expression of hormone receptor and there is significant cross talk between them.^29,292,293^

Endocrine therapy combined with double blocking HER2 therapy significantly improved PFS, which may be considered for the selection of HER2+/HE+ breast cancer patients.^273,294^ The randomized clinical trials showed its efficacy to be inferior to that of chemotherapy plus anti-HER2 regimens. For co-expression of HER2 and hormone receptors patients, combination strategy of aromatase inhibitors (AIs) with anti-HER2 therapies has also been suggested as a new therapeutic option.^295–297^ Some phase III trials have explored the superiority of dual blockage, as compared with the single antihormonal therapy.^298^ Besides, chemotherapy-free strategies have always been recommended. TAnDEM study,^299^ the first randomized phase III trial, enrolled 207 patients of HER2− and hormone receptor-positive MBC, without brain metastases and prior chemotherapy. These patients were randomized to receive either anastrozole alone or AI combined with trastuzumab. The primary efficacy point of PFS was found to be extended by 2.4 months in the combination arm (4.8 vs 2.4 months; *p* = 0.0016). OS was not statistically improved in the total and hormone receptor-positive populations. Importantly, 70% of patients in the anastrozole-alone arm crossed over to receive trastuzumab after progression. Thus, resulting in nearly 15% patients of combination arm experiencing 2-year RFS, suggesting that this regimen was appropriate for a small portion of patients.^300^

In safety analysis, incidence of grade 3 and 4 AEs was 28% in the combination arm and 15% in the anastrozole-alone arm, thus showing that serious AEs were more frequent with the combination therapy.^299^

Small eLEcTRA trial^297^ had similar design and enrollment criteria, however, letrozole was used instead of anastrozole in this study. The authors reported an improvement trend in TTP (14.1 vs 3.3 months; *p* = 0.23). These studies support the use of HER2-targeted therapy combined with nonsteroidal AIs, as a valid effective chemotherapy-free option in the treatment of triple-positive, postmenopausal patients.

The EGF30008 trial enrolled patients with HR+/HER2+ MBC, but not HR+/HER2-disease to compare letrozole alone or additional use of lapatinib.^301,302^ From the total of 1286 patients, 219 HER2-positive cases were randomized in this study, without prior therapy for MBC. Compared with letrozole and placebo, a significant improved PFS has been seen in combination of letrozole and lapatinib (8.2 vs 30 months; *p* = 0.019). No OS benefits were seen significantly, and the incidence of AEs was higher in add-on, than that with letrozole alone (8% vs 4%, *p* < 0.05).

#### HER2-positive CNS metastases

The CNS metastases have special clinical features.^303,304^ As seen in previous meta-analysis, HER2-positive patients who receive trastuzumab adjuvant therapy, compared with chemotherapy alone, may have a higher risk of brain metastasis as an initial recurrence site.^305–307^ Traditional surgical and radiotherapy for the treatment of brain metastases from breast cancer have great limitations and safety risks.^308,309^ While, chemotherapy and targeted therapy, as a broad and relatively low-toxicity treatment method, has attracted increased attention.^310–312^ As the molecular structure prevents thing crossing of the blood–brain barrier, most of the currently available anti-HER2 drugs, like trastuzumab, pertuzumab and ado-trastuzumab, have limitations to treat such brain metastases.^309,313^ Thus, small molecules, such as TKI, may provide better option to achieve therapeutic concentrations within the brain sanctuary.^118,313–315^

As lapatinib possess an ability to penetrate the blood–brain barrier, this small molecular TKI agent has been explored for its potential efficacy in CNS metastases for HER2-positive MBC, after the first-line trastuzumab-based treatment progression.^118,316,317^ In a study of 39 patients heavily pretreated with trastuzumab plus taxanes chemotherapy, having progression despite radiation, two patients achieved a PR evaluation, and five experienced at least a 30% volumetric reduction in progressive CNS disease. The potential efficacy of lapatinib alone in trastuzumab-resistant brain metastases needs further evaluation in larger scale cohorts.^119,318^ Lapatinib was approved by the FDA in 2007 for its use in combination with capecitabine for the treatment of HER2-positive MBC that had progressed despite standard treatment. The combination of lapatinib and capecitabine has been correlated with a lower rate of CNS relapse, compared with capecitabine alone.^120,257,314,319^ Nonetheless, the CEREBEL study^320^ showed no difference (*p* = 0.360) in the incidence of CNS recurrence as a first site of disease progression between lapatinib–capecitabine group (3.0%) and trastuzumab–capecitabine group (5.0%). Safety analysis reported that serious AEs in the lapatinib–capecitabine and trastuzumab–capecitabine groups were 13% and 17%, respectively. The major endpoints of PFS and OS were inconclusive and no differences in the incidence of CNS metastasis were observed between the two groups. The efficacy of lapatinib–capecitabine group could be related to the previous trastuzumab treatment, and/or the number of treatment lines selected in the context of metastatic disease.^303,306,311,321^

As per the Anatolian Society of Medical Oncology review, lapatinib plus capecitabine was recommended for HER2-postive ABC patients with brain metastasis.^120^ A total of 203 patients with HER2-positive MBC, who had progressed after trastuzumab-containing chemotherapy, were retrospectively evaluated at 11 centers between September 2009 and May 2011. All patients had undergone cranial radiotherapy and lapatinib therapy, initially. Median follow-up was 10.5 months (range 1–38 months). The total RR was 27.1%, including two cases of CR (2.4%) and 21 cases of PR (24.7%). The median PFS was 7 months (95% CI, 5–9), and the median OS was 13 months (95% CI, 9–17). Grade 3–4 AEs were hand and foot syndrome (9.4%), diarrhea (8.3%), fatigue (5.9%), and rash (4.7%). There were no symptoms of cardiac events. The combination of lapatinib with capecitabine used had good efficacy and tolerance.^322^

A French phase II, randomized study assessed the initial treatment of capecitabine, combined with lapatinib, in HER2-positive patients with diffuse CNS metastases, along with whole brain radiotherapy (WBRT).^309,317^ This study showed that capecitabine and lapatinib combined regimens had an impressively high ORR = 66%, as the initial method. The regimen was effective for both CNS and extra-CNS diseases and delayed WBRT by an average of 8 months.^115^

The LANDSCAPE study^323^ was a single arm, phase II, open-label, multicenter study, that enrolled 44 previously untreated patients, with brain metastases from HER2-positive breast cancer. The tumor volume shrunk by at least half in nearly two-third of these patients after the treatment with lapatinib combined with capecitabine. Lapatinib plus capecitabine group had an ORR of 65.9% (95% CI, 50.1–79.5) with all partial responses, median PFS of 5.5 months, and median OS of 17 months. This study makes it possible for lapatinib combined with capecitabine to replace WBRT as the first-line treatment for brain metastasis of HER2-positive breast cancer. Moreover, a larger scale randomized phase III trial is warranted.^313^

The need of treatment and prevention of brain metastasis in breast cancer patients has yet not been met. Current systemic treatments lack standard care.^319,324^ The first clinical decision on systemic therapy should be based on the best choice for systemic disease management outside the CNS. In previous retrospective studies, T-DM1 seemed to be an effective and well-tolerated treatment for brain metastasis in HER2-positive breast cancer patients.^325,326^ These findings require a prospective validation. Thus, clinical trials of new drugs related to the CNS remains a high priority.^313–315^

#### Emerging treatment strategies for HER2-positive MBC

Discovery of new targets will lead to development of new and more effective drugs for the treatment of HER2-positive breast cancer.^281^ Data from several interesting studies of HER2-positive MBC were presented at 2018 ASCO annual meeting.^327^

Early phase studies using novel antibody-drug conjugates (ADCs), such as trastuzumab-deruxtecan^175,328–330^ suggest that these drugs are relevant to the clinical activity of pretreated patients; in addition, these ADCs may also play a role in tumors with low HER2 expression. These trials, while not immediately changing clinical practice, indicate the future direction of drug development in this area. T-DM1 in combination with neratinib,^331^ a TKI, produced high response rates. A comprehensive analysis of two tucatinib studies showed that after local treatment of brain metastases, systemic therapy is effective if the CNS progresses in isolation and extracranial disease is stable.^332,333^

Anti-HER2 antibodies have synergistic effects with PD-L1 inhibitors.^334^ In the exploratory study of immune checkpoint inhibitors for HER2-positive ABC, pembrolizumab in combination with trastuzumab has demonstrated benefit efficacy and tolerate safety in trastuzumab-resistant patients with PD-L1 positive detection. PANACEA study^335^ showed that 15% ORR can be obtained with trastuzumab plus Pembrolizumab for treatment of HER2+ MBC after drug resistance, providing an initial basis for combined anti-HER2 immunotherapy. The results of this study provide us with some beneficial enlightenment. Currently, a number of phase III trials are exploring combined immunotherapy against HER2. Further studies should focus on this breast cancer subtype with PD-L1 positive individuals as initial treatment and choose the right subgroup and the right combination strategies.^334,336,337^

CDK4/6 inhibitors affect cell cycle and are potentially complementary to trastuzumab.^174^ Combination regimens have also been observed to delay tumor growth in phase Ib clinical trials. PATINA and MONARCHER are exploring anti-HER2 therapy in combination with CDK4/6 inhibitors.^338^

## Conclusions and future perspectives

Breast cancer, which affects many women worldwide, is a complex and heterogeneous disease, which can divided into many subtypes. The HER2-positive breast cancer accounts for 20–25% of all breast cancers, and it is of great concern in research and clinical practice. This highly malignant cancer is very aggressive and has poor metastasis and recurrence outcomes. Anti-HER2 therapy is the cornerstone for early and advanced HER2-positive breast cancer. All kinds of regimens for HER2-positive breast cancer should follow anti-HER2 principle.

Trastuzumab is a landmark drug for the anti-HER2 treatment. It changes the treatment pattern and prognosis in HER2-positive breast cancer patients. One-year treatment with trastuzumab is a standard for the adjuvant therapy. Extending the treatment time has been confirmed not to further improve the efficacy, while it may increase the cardiotoxicity events. However, shortening the treatment period of trastuzumab administration shows no benefits, to some extent. Pertuzumab showed an overall good efficacy in adjuvant therapy. High-recurrence risk groups (positive-lymph nodes or ER/PR negative patients) can benefit significantly from double-targeted adjuvant therapy. At present, this innovative treatment scheme has been recommended in multiple guidelines in accordance with experts in China and abroad.

Nevertheless, 25% of early HER2-positive breast cancer patients still experience disease recurrence after initial anti-HER2 therapy. Currently, the standard of first-line care for HER2-positive MBC is dual anti-HER2 blocking with pertuzumab and trastuzumab plus chemotherapy. The T-DM1 is recommended as the second-line treatment, and small-molecule TKI as the third-line.

Although trastuzumab, pertuzumab, lapatinib, and neratinib are greatly promising drugs, some patients may show no response or develop drug resistance after a period of treatment. The emergence of many new drugs provides a new view for combined treatment strategies against HER2. In clinical practice, it is a direction of future clinical research to explore new clinical trial methods, especially research design on gene level. In the next 10 years, the detection techniques of HER2 will be further refined and the results will be more accurate. Research on molecular biology of breast cancer should be carried out to discover the key genes that affect the proliferation and metastasis of breast cancer cells. More convenient and valid prediction of prognostic factors will guide the individualized diagnosis and treatment of HER2-positive breast cancer. Through a large number of data analysis, revealing the law of efficacy and safety to determine reasonable administration of regimens should be our persistent effort in the struggle for ultimate cure and greater survival benefits.

## Supplementary information

Abbreviation Form

Certificate_of_editing-ENLMD_9
