# Supplementary material for: Targeted therapeutic options and future perspectives for HER2-positive breast cancer
Source: Signal Transduct Target Ther. 2019 Sep 13;4:34. doi: 10.1038/s41392-019-0069-2 (PMC6799843; doi:10.1038/s41392-019-0069-2)
Supplement: Supplementary file 1 — Abbreviation Form [file 41392_2019_69_MOESM1_ESM.pdf]

Abbreviations and Acronyms

ABC Advanced breast cancer

ADCs Antibody -drug conjugates

ADCC Antibody-dependent cell-mediated cytotoxicity

AEs Adverse events

AIs Aromatase Inhibitors

ALTTO Adjuvant Lapatinib and/or Trastuzumab Treatment Optimization

ASCO American society of clinical oncology

ASMO Anatolian Society of Medical Oncology

BCIRG Breast Cancer International Research Group

BOLERO-1 Breast Cancer Trials of Oral Everolimus-1

BOLERO-3 Breast Cancer Trials of Oral Everolimus-3

CEP17 Chromosome-17 Polysome

cfDNA Cell-free DNA

CHF Congestive heart failure

CISH Chromogenic in situ hybridization

CI Confidence interval

CNS Central nervous system

CBR Clinical benefit rate

CR Total relief

CTC Circulating tumor cells

CTCAE Common Terminology Criteria for Adverse Events

DCR Disease control rate

DLTs Dose limiting toxicities

EBC Early breast cancer

ECD Extracellular domain

ELISA Enzyme-Linked Immunosorbent Assay

ExteNET Extended Adjuvant Treatment of Breast Cancer with Neratinib

FinHer Finland Herceptin

FISH Fluorescence in situ hybridization

HER2 Human epidermal growth factor receptor 2

HORG Hellenic Oncology Research Group

HR Hazard ratio

iDFS Invasive disease-free survival

IHC Immunohistochemical

LVEF Left ventricular ejection fraction

MBC Metastatic breast cancer

NCI National Cancer Institute

NCCN National Comprehensive Cancer Network

NSABP National Surgical Adjuvant Breast and Bowel Project

ORR Objective Response Rate

OS Overall survival

pCR Pathologic complete response

PD Progressive disease

PFS Progression free survival

RCT Randomized control trial

PR Partial relief

RFS Recurrence free survival

RT-PCR Reverse transcription polymerase chain reaction

RR Relative risk

SABCS San Antonio Breast Cancer Symposium

SD Stable disease

SEER Surveillance, Epidemiology, and End Results

SISH Silver in situ hybridization

TEACH Tykerb Evaluation after Chemotherapy

T-DM1 Trastuzumab-maytansine

TKIs Tyrosine kinase inhibitors

TTP Time to disease progression

U.S. FDA United States Food and Drug Administration

VEGF Vascular endothelial growth factor

WBRT Whole brain radiotherapy
