# Supplementary material for: Targeted therapeutic options and future perspectives for HER2-positive breast cancer
Source: Signal Transduct Target Ther. 2019 Sep 13;4:34. doi: 10.1038/s41392-019-0069-2 (PMC6799843; doi:10.1038/s41392-019-0069-2)
Supplement: Supplementary file 2 — Certificate_of_editing-ENLMD_9 [file 41392_2019_69_MOESM2_ESM.pdf]

CERTIFICATE OF ENGLISH EDITING

This document certifies that the paper listed below has been edited to ensure that the language is clear and free of errors. The edit was performed by professional editors at Editage, a division of Cactus Communications.The intent of the author's message was not altered in any way during the editing process. The quality of the edit has been guaranteed, with the assumption that our suggested changes have been accepted and have not been further altered without the knowledge of our editors.

Title Of The Paper

Targeted therapeutic options and future perspectives for HER2-positive breast cancer

Authors

Jiani Wang, Binghe Xu *

Job Code

ENLMD_9

editage by CACTUS

Signature

Vikas Narang

Vikas Narang,
Senior Vice President,
Operations-Author Services, Editage

Date of Issue
April 25, 2019

Editage, a brand of Cactus Communications, offers professional English language editing and publication support services to authors engaged in over 500 areas of research. Through its community of experienced editors, which includes doctors, engineers, published scientists, and researchers with peer review experience, Editage has successfully helped authors get published in internationally reputed journals. Authors who work with Editage are guaranteed excellent language quality and timely delivery.

INFORMATION SECURITY MANAGEMENT SYSTEM DNV·GL ISO/IEC 27001

CACTUS.

Contact Editage

Worldwide	Japan	Korea	China	Brazil
request@editage.com	submissions@editage.com	submit-korea@editage.com	fabiao@editage.cn	contato@editage.com
+1 877-334-8243	+81 03-6868-3348	1544-9241	400-005-6055	0800-892-20-97
www.editage.com	www.editage.jp	www.editage.co.kr	www.editage.cn	www.editage.com.br
